# DIFFERENCES IN LIFE COURSE PATHWAYS TO DEMENTIA INCIDENCE FOR BLACK AND WHITE OLDER AMERICANS

**DOI:** 10.1093/geroni/igac059.216

**Published:** 2022-12-20

**Authors:** Mateo Farina

**Affiliations:** University of Southern California, Los Angeles, California, United States

## Abstract

Black Americans are 2-3 times more likely to have dementia than White Americans. Studies have evaluated how differences in exposures to life course risk factors, such as level of education, may be contributing to this disparity. When using this approach, substantial reductions in cognitive health inequality across race were accounted for, but not completely explained. However, this research assumes an underlying similarity in life course pathways to cognitive impairment risk across race groups. We used longitudinal data from the Health and Retirement Study (2000-2016) to evaluate life course pathways to dementia risk across race groups. We find substantial differences across race groups. Dementia risk for older black adults is greatly shaped by southern birth and education. For older white adults, adulthood factors such as wealth, health behaviors, and cardiometabolic conditions has a greater role. Our findings support calls for a closer examination of within group analysis among minoritized populations.

